# Auditory evoked potentials and suicidal behaviors in patients with major depressive disorders

**DOI:** 10.1038/s41598-021-86602-7

**Published:** 2021-03-31

**Authors:** Ji Sun Kim, Sungkean Kim, Ho-Sung Lee, Young Joon Kwon, Hwa Young Lee, Se-Hoon Shim

**Affiliations:** 1 Department of Psychiatry, College of Medicine, Soonchunhyang University Cheonan Hospital, 31 Suncheonhyang 6-gil, Dongnam-gu, Cheonan, 31151 Republic of Korea; 2 Department of Human-Computer Interaction, Hanyang University, Ansan-si, 15588 South Korea; 3 Department of Pulmonology and Allergy, Cheonan Hospital, Soonchunhyang University, Cheonan, Republic of Korea

**Keywords:** Psychiatric disorders, Neuroscience

## Abstract

Loudness dependence of auditory evoked potentials (LDAEP) has been proposed as a biological marker of central serotonergic activity related to suicides. This study’s objective was to analyze the difference in LDAEP between depressed patients with suicide attempts (SA) and suicidal ideation (SI). It included 130 participants (45 depressed patients with SA, 49 depressed patients with SI, and 36 healthy controls) aged > 18 years who exhibited LDAEP during electroencephalography. Psychological characteristics and event-related potentials of the three groups were compared. There was no significant difference in LDAEP between major depressive disorder (MDD) patients with SA and SI (*p* = 0.59). MDD patients with SI, who attempted suicide had significantly lower LDAEP than healthy controls (*p* = 0.01 and *p* = 0.01, respectively). However, the significance disappeared when psychological characteristics were controlled. Our results suggest that LDAEP might not be possible biomarkers for suicidal behaviors in patients with MDD. Further studies to assess the biological basis of suicide and identify the underlying dimensions that mediate the relationship between the biological basis and suicidal behaviors will be needed.

## Introduction

According to the World Health Organization’s statistics, approximately 800,000 individuals commit suicide every year, which is one every 40 s. Suicide is a global phenomenon and occurs throughout the lifespan. Affective disorders are known to be one of the leading causes of suicide^1^. Since effective methods for predicting suicide attempts (SA) are presently unavailable, tools for effective and evidence-based suicide prevention interventions need to be developed.

Although most individuals with suicidal ideation (SI) never make a SA^2,3^, there is few scientific research to distinguish suicide attempters from those intending suicide but not attempting it^2,4^. This gap has resumed the necessities for studies on suicide within an ideation-to-action framework, with emphasis on finding the factors that promote the transition from intending suicide to SA^2,4–9^.

A plenty of studies have been conducted to find the biological basis of suicide and the studies revealed the changes of the serotonergic function have been associated with SA^9^. Postmortem studies have shown that death by suicide is related to lower serotonin transporter binding in the orbital prefrontal cortex with higher 5-HT1A and 5-HT2A receptor binding in the dorsolateral prefrontal cortex^10^. In addition, the lower 5-hydroxyindoleacetic acid (5-HIAA) in the CSF have been related to SA in various psychiatric illness^11^. Imaging studies have also reported that changed serotonergic function affects suicidal behavior, suggesting a lower serotonin transporter binding in the frontal regions of impulsive participants^12^. The applying results from postmortem studies is limited by the fact that they are not generalized in living persons, and no formal consensus exists in relating a neurobiological mechanism with suicide^13^. However, the previous studies tend to meet at a univocal consensus only for the serotoninergic system, particularly with respect to the polymorphism of the serotonin transporter (5-HTTLPR) gene^13^. Although the relationship between the low 5-HT neurotransmission and suicide still remain unspecific^14^, many studies have indicated to serotonergic activity as possibly the most related markers for suicide^9,13,15^.

Loudness dependence of auditory evoked potentials (LDAEP) is an event related potential (ERP) parameter used to assess changing types of the N1-P2 amplitude related to varying loudness levels of auditory stimulation^16^. It is known to be an indicator of auditory cortex activity that is mediated via serotonergic neurons^17^ and implies individual differences in cortical sensory processing associated with serotonin^17^. The slope of the N1/P2 component is more gentle when the central serotonergic activity is strong, and vice versa^17,18^. Related to the above association, LDAEP is stronger in major depressive disorder (MDD) patients than in healthy controls^19,20^. Besides its association with 5-HT activity depending on the LDAEP, previous researches revealed stronger LDAEP in more impulsive individuals^21^, suggesting behavioral inhibition^22^. Previous studies also reported that participants who were sensitive to external stimuli exhibited higher emotional responses^23^. In this context, a specific association between LDAEP and emotional sensitivity has also been reported^22^. In contrast, the various previous studies proposes that the LDAEP dose not have enough sensitivity and specificity to acute alterations in serotonergic neurotransmission^24^. The recent review concluded that the LDAEP could be a potential predictor of antidepressant treatment response, but the findings do not underpin for its usefulness as a marker of central serotonergic function^24^. A plenty of studies have cast doubts on the utility of LDAEP as a biomarker, citing no treatment effect on LDAEP or non-specificity of LDAEP for serotonin activity^25–27^, despite the 5-HT system having clinical importance in various psychiatric illness^24,28^. Despite of the doubts on the relation between SA and LDAEP, a recent PET study revealed that.

exploratory analysis showed multiple regions in which LDAEP significantly correlated with 5-HT1A throughout the brain in male population^29^. Considering that several previous postmortem studies reported that 5-HT1A receptor binding was increased or decreased in suicide victims^30–33^, the questions for the relation between LDAEP and suicide still remains.

Meanwhile, Uhl and his co-workers revealed significant changes concerning LDAEP^21^, but their follow up study did not show significant differences in LDAEP between the groups with and without SA^34^. With Uhl’s null study, the authors pointed out that further studies would be necessary to detect and describe further influences on serotonergic function and confounding factors like medication, smoking, age, gender, comorbidities and methods of suicidal attempts^21^. In this context, further studies, including a group of suicidal behaviors with a larger sample size and considering the confounding factors are needed to verify the utility of LDAEP as a biomarker for suicide related to 5-HT activity.

Besides serotonergic activity, a number of potential candidate biological and clinical endophenotypes for suicidal behaviors, based largely on their association with the phenotype^13,35^ have been identified. Impulsive-aggressive traits have been suggested as a possible important endophenotype^13,36,37^. Emotional sensitivity and poor impulse control are shown in patients with suicidal behavior^38,39^. Previous studies revealed that LDAEP could reflect emotional sensitivity and impulsivity^22^.The serotonergic systems and impulsive traits could play a critical role in suicide^40^, suggesting that LDAEP slopes might differ quantitatively among patients with SA, as compared with those having SI and healthy controls. Besides the serotonergic system, regarding the sensory stimulus processing in relation to symptoms of depression and suicide, LDAEP changes related to suicide behaviors need to be evaluated. Previous studies demonstrated that lower LDAEPs decreased responsiveness for exogenous stimuli and a weak loudness dependency reflected lower exogenous attention^41,42^. Zuckerman focused on the relation between stimulus intensity dependence and personality factors like impulsiveness, aggressiveness and sensation seeking behavior^27,43^. Considering that the above personality factors were often observed in patients with suicide behavior^44–46^, we hypothesized that LDAEP would be different between patients with SA and SI.

Despite a plausible relationship between LDAEP and depression with suicidal behavior^22^, research investigation is yet to be concluded on the relationship among LDAEP, SA, and SI in depressed patients. While several studies have expressed doubts on the utility of LDAEP as a marker of central 5-HT function, some studies have reported that patients with SA showed higher LDAEP and decreased serotonin activity^47,48^. Furthermore, the results could not be confirmed due to the small sample size, varying duration levels between the SA and EEG test, and the various frequencies of fatality in SA. To elucidate suicidal behaviors, an electrophysiological marker is needed to distinguish SA from SI. In addition, to evaluate the possible role of LDAEP in suicidal behaviors, studies using larger sample sizes of the population are urgently needed to evaluate post-suicidal outcomes.

Hence, this study’s purpose was to assess the dissidences in LDAEP among patients with MDD combined with SA, those with SI alone, and healthy controls. In addition, we compared their various clinical characteristics, such as impulsivity and emotional dysregulation. We hypothesized that clinical characteristics such as difficulties in emotional regulation and impulsivity, and LDAEP differ between depressed patients with SA, those with SI, and healthy controls. This study was performed to demonstrate the association between LDAEP and suicidal behaviors reflecting emotional dysregulation and impulsivity.

## Results

### Participants

Tables 1 and 2 present baseline demographic and clinical characteristics of patients diagnosed with MDD along with SA, SI, and healthy controls. There were no significant group differences according to age and gender (Table 1). The healthy control group had significantly higher education than depressed patients with SA (*p* = 0.003).

**Table 1:** Comparison of baseline demographic data among MDD patients with suicide attempts and suicide ideation, and healthy controls.

	Suicide attempt (N = 45)	Suicide ideation (N = 49)	Healthy controls (N = 36)	*p*
	Mean ± SD or N (%)			
Age (years)	32.07 ± 9.49	32.06 ± 9.34	31.44 ± 5.20	0.933
**Gender**				
Male	20 (44.4)	23 (46.9)	19 (52.8)	0.750
Female	25 (55.6)	26 (53.1)	17 (47.2)	
Education (years)	12.44 ± 1.58	12.98 ± 1.91	13.72 ± 2.15	0.011 ^a^

^a^With suicide attempt vs. with suicide ideation, p = 0.169; with suicide attempt vs. healthy control, p = 0.003; with suicide ideation vs. healthy control, p = 0.073.

**Table 2:** Comparison of baseline clinical symptoms among MDD patients with suicide attempts and suicidal ideation, and healthy controls.

	Suicide attempt (N = 45)	Suicide ideation (N = 49)	Healthy controls (N = 36)	*p*	*Pairwise test* *P*
	Mean ± SD or N (%)				
Clinical symptom characteristics					with attempt vs. with ideation
Beck depression inventory	54.22 ± 12.12	54.65 ± 9.63	25.33 ± 4.67	< 0.001	0.735
Beck anxiety inventory	26.91 ± 14.99	35.43 ± 14.24	4.19 ± 9.34	< 0.001	0.003
Difficulties in emotion regulation scale	110.40 ± 24.57	115.59 ± 26.81	66.03 ± 13.73	< 0.001	0.249
Barrett impulsivity scale	75.29 ± 9.72	76.16 ± 11.74	64.36 ± 10.49	< 0.001	0.554
Attentional impulsivity	19.69 ± 3.75	20.59 ± 3.97	16.22 ± 3.62	< 0.001	0.212
Motor impulsivity	25.38 ± 4.51	25.67 ± 5.48	24.31 ± 4.02	0.436	0.628
Non-planning impulsivity	30.22 ± 3.98	29.90 ± 4.64	23.50 ± 4.30	< 0.001	0.774

### Clinical symptoms

In terms of clinical symptoms, MDD patients with SI showed significantly higher Beck Anxiety Inventory (BAI) scores than MDD patients with SA. The results revealed no significant differences between MDD patients with SI and those with SA based on scores of the Beck Depression Inventory (BDI), Difficulties in Emotion Regulation Scale (DERS), Barratt Impulsiveness Scale (BIS-11), and BIS subscales (Table 2). Compared with healthy controls, MDD patients with SA and SI showed significantly higher scores in the BDI, BAI, DERS, and BIS scores.

### Loudness dependence auditory evoked potentials (LDAEP)

**Figure 1:**
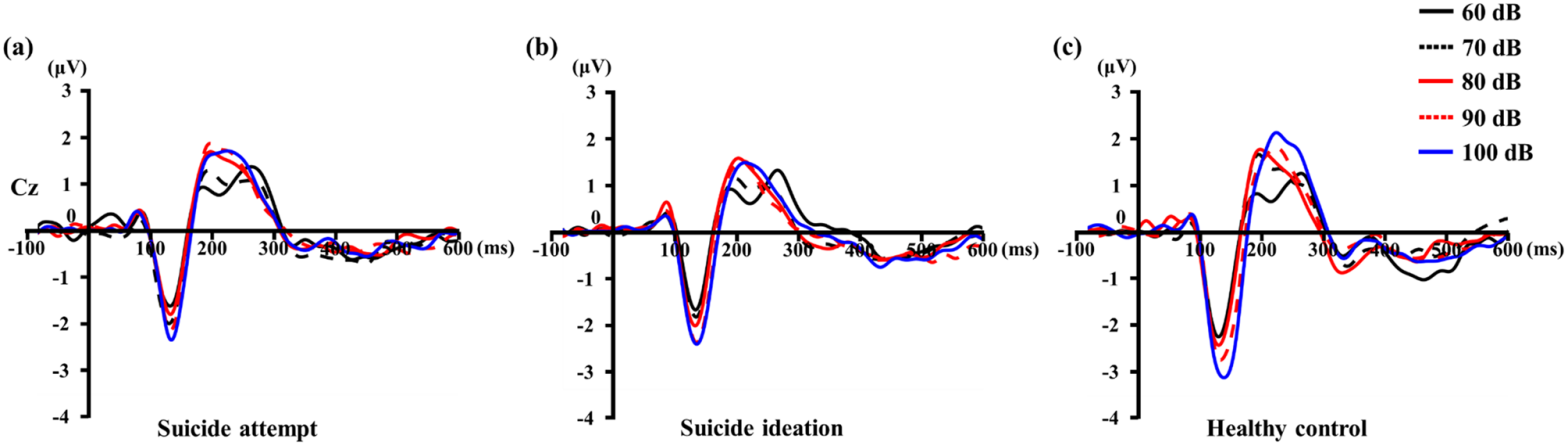
Grand average of loudness dependence of the auditory evoked potentials (LDAEP) event-related potentials (ERPs) at the Cz electrode for major depressive disorder (MDD) patients with: suicide attempts (**A**) and suicidal ideation (**B**), and healthy controls (**C**). The auditory stimuli were generated with E-Prime software.

**Figure 2:**
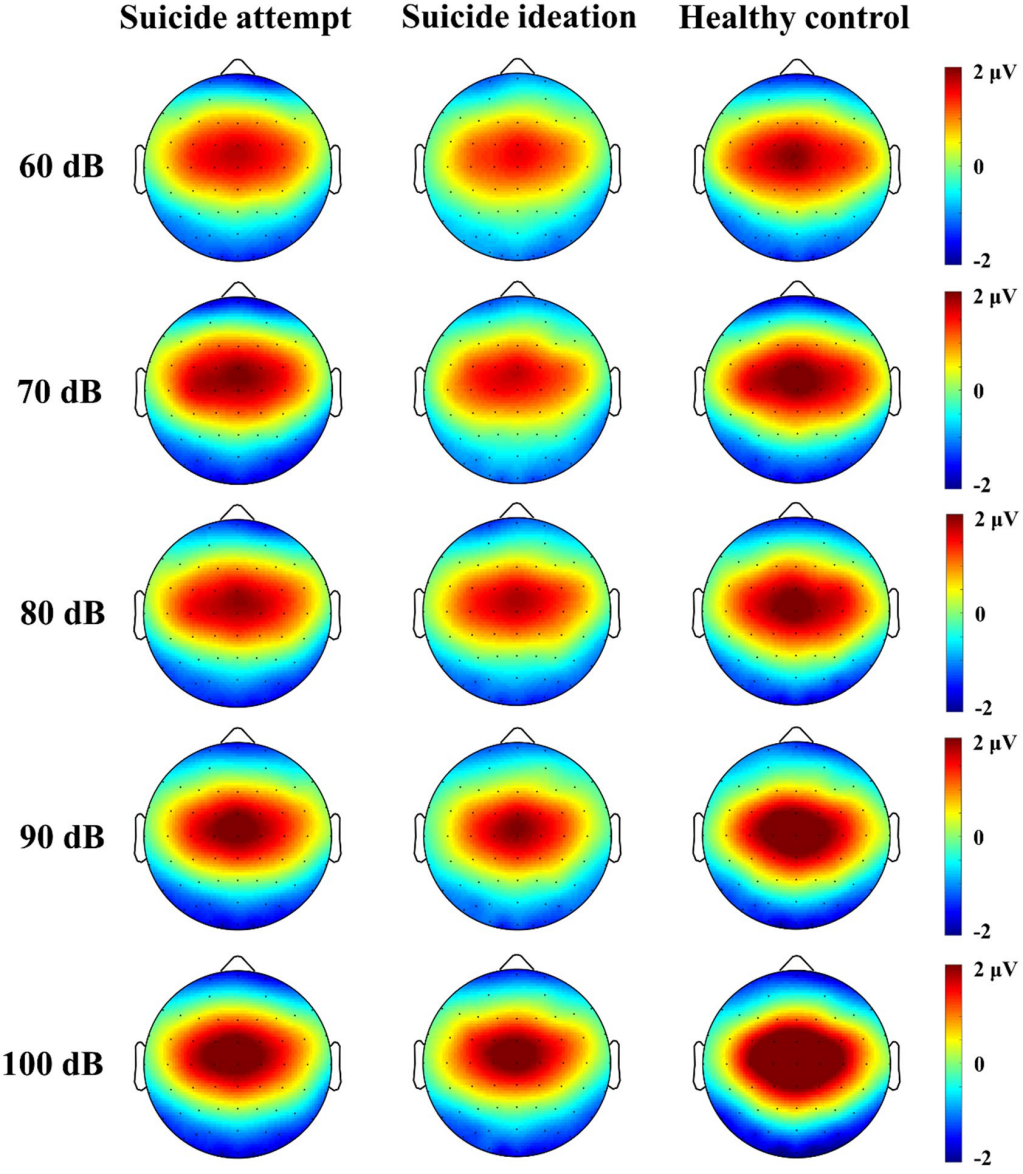
Topographic maps of P2 component minus N1 component for each auditory stimulus in MDD patients with suicide attempts and suicide ideation, and healthy controls.

The three groups of subjects showed significantly different LDAEP levels at the Cz electrode (F = 3.37, *p* = 0.04). However, there was no significant difference of LDAEP levels at the FCz and Cz electrodes (F = 0.81, *p* = 0.92 and F = 1.08, *p* = 0.34, respectively). MDD patients with SA had significantly weaker LDAEP than healthy controls (0.16 ± 0.30 and 0.36 ± 0.39, respectively, with *p* = 0.01). In addition, significantly different LDAEP were found among MDD patients with SI as compared with healthy controls (0.20 ± 0.39, *p* = 0.01). However, the significance disappeared when clinical variables such as BDI, BAI, BIS, and DERS were controlled. There was no significant difference in LDAEP between MDD patients with SA and SI (p = 0.59). The grand average of the LDAEP at the Cz electrode for each group is shown in Fig. 1. Topographic maps of P2 component minus N1 component for each auditory stimulus in MDD patients with suicide attempts and suicide ideation, and healthy controls are shown in Fig. 2.

## Discussion

This study investigated whether LDAEP varied in depressive patients with SA and SI, and healthy controls. First, there was no significant difference in LDAEP between MDD patients with SA and SI. Second, depressed patients with SA and SI showed weaker LDAEP than healthy controls, however, the changes disappeared when clinical variables were controlled. Third, there was no significant correlation between LDAEP and psychological scale scores in depressed patients with SA.

Unexpectedly, emotional regulation difficulties and impulsivity did not show difference between depressed patients with SA and SI. Although emotional regulation difficulties and impulsivity traits were important clinical characteristics of suicide attempters^38,39^, they might be a cause of suicidality for some, but not all, disorders. A recent study demonstrated that emotional regulation difficulties did not independently predict suicidal behaviors^38^. The other possible explanation is that the self-report could not reflect the genuine characteristic of emotional regulation difficulties and impulsivities in suicide attempters. Considering that the suicidal behaviors are variably associated with different risk factors and have aetiological heterogeneity^49,50^, the other clinical manifestation might affect our study population with SA.

In addition, the difference in LDAEPs was also not observed between depressed patients with SA and SI. The first possible explanation could be related to the possibility of LDAEP as a biomarker for suicide. Many previous studies showed inconsistent findings on the role of LDAEP and also had doubts on its utility as a marker of central 5-HT function^24^. Additionally, composing various inconsistent results to present a significant model explaining the role of serotonin in suicide^40^ is challenging. Although various evidences suggest the important role of serotonin in suicide, there is no consistency between the previous results. Apparently, genetic and epigenetic factors play a critical role in the molecular mechanisms underlying the individual risk of suicide^40^. Additional studies are needed to investigate the role of other neurotransmitters implicated in suicide such as dopamine, norepinephrine and glutamate, since their interaction with serotonin yielded that abnormalities in serotonin alone are unlikely to satisfactorily explain the complex phenomenon of suicide. To summarize, LDAEP might not be a biomarker for suicide behaviors, and our findings corroborate the previous study by Uhl and his co-workers that revealed no significant differences in LDAEP between the groups with and without SA^21,34^. The present study might be meaningful in that confirmed the Uhl’s null data even after controlling confounding factors like medication, smoking, age, gender, and methods of suicidal attempts.

Assuming that LDAEP reflects the activity of 5-HT, the second possible explanation is that it might not be changed by the presence or absence of SA, but modified by illness or diseases, such as bipolar or unipolar depression. The enzymes for the biosynthesis of both norepinephrine and serotonin are reduced in the locus ceruleus of bipolar depression patients committing suicide, but not in unipolar depression patients who commit suicide^51^. Thus, the level of serotonin activity may not differ quantitatively with or without SA, but vary with the type of disease. The last possible explanation for the lack of difference in LDAEP between those with SA and SI is the unfixed nature of LDAEP levels. Since LDAEP is not fixed in one’s lifetime; the serotonergic activity in psychiatric diseases might be more sensitive to changes in serotonin-dependent states such as mood or impulsivity^21^, which may have been the case in this study’s suicidal patients, who were diagnosed with depression. In this regard, Uhl and co-workers detected similar low LDAEP levels immediately after the SA (days 2 and 5) and at a later date (day 16), but a higher LDAEP, i.e., lower serotonergic activity on day 9. Given that this study used an EEG to evaluate patients within 7 days of their SA; the immediate and delayed EEGs might have been similar and resembled the state of depression. Thus, due to changes in LDAEP based on test dates, its levels might differ between depressive patients with SA and SI, and its unfixed nature might reflect its lack of utility as a biomarker for suicide.

While this study’s MDD patients with SA and SI had significantly weaker LDAEPs than healthy controls; the changes disappeared when clinical variables were controlled. This suggests that LDAEP differences between depressed patients with suicidal behaviors and healthy controls might not be due to the clinical characteristics of suicidal behaviors, but a reflection of the innate differences between the two groups. Compared with previous studies reporting an inverse correlation between LDAEP and central serotonergic activity^52^, our results that MDD patients exhibited a lower LDAEP were not in line with earlier studies which had indicated different LDAEP levels among MDD patients and healthy controls^19,53^. Additionally, many previous studies had reported that patients with MDD showed higher LDAEP and lower serotonin activity than healthy individuals^19,53^. Drevets and his co-workers reported that the binding potential of 5-HT1A receptors in the raphe and mesiotemporal cortex of unmedicated subjects with MDD was lower than in the controls^54^. A previous study presented electrophysiological evidence suggesting that LDAEP was higher among unmedicated MDD patients with SA than in their depressive counterparts who did not attempt suicide^48^. Further, Chen and his collegues reported that depressive patients who manifested acute SA showed higher LDAEP than healthy individuals^47^.

However, in another PET study that used the same radioligand, MDD patients undergoing treatment with antidepressants exhibited greater 5-HT1A receptor-binding potential in their raphe and mesiotemporal cortex than controls and antidepressant-naïve patients with MDD^55^. Similarly, other studies also reported no differences or a weaker LDAEP in patients with MDD compared with healthy individuals^21,23,27^. Uhl and his colleagues reported that depressed patients with a history of SA exhibited a weak LDAEP^21^. In addition, the first study which evaluated LDAEP immediately after SA, found the LDAEP weaker in non-suicidal depressed patients than in healthy individuals^34^. In contrast, previous studies’ findings were not consistent regarding SA by MDD patients. These inconsistent results on LDAEP in relation to suicidal behaviors reflect that it might not be a possible biomarker for suicide, but for characteristics of MDD, such as depressed mood or emotional dysregulation. Additionally, a weaker LDAEP in depressed patients with SA and SI is also explained by the correlation between LDAEP and various clinical manifestations of depression. A previous study revealed that a weak LDAEP was indicative of a worse treatment response to Selective Serotonin Reuptake Inhibitor (SSRIs), such that a very low LDAEP could induce treatment resistance^56^. Since SA and SI represent factors for poor prognosis of depression and refractory depression^57,58^; this study’s finding of a lower LDAEP in those with depression and suicidal behaviors is plausible.

These findings relating to no significant differences in LDAEP between depressed patients with SI and SA, and healthy controls through controlling psychological variables suggest that specific alterations in LDAEP cannot generally be expected in MDD. In addition, although LDAEP might not be suitable as a biomarker for MDD diagnosis, it may serve as a possible biomarker for behavioral phenotypes—emotional regulation or poor impulse control—affecting suicidal behaviors. Since this study also demonstrated no significant correlations between LDAEP and psychological scale scores in depressed patients with SA; it cannot reveal the intermediated phenotype linked to suicidal behaviors. A possible explanation for the null findings without significant differences in DERS and BIS scores between depressed patients with suicidal thoughts and healthy controls, could be the inability of our self-reported indices to reflect the neural/cognitive basis of suicide. To reflect suicide or poor impulse control explicitly, other effective methods (e.g. laboratory behavioral measures such as GoNogo or two choice impulsivity paradigm) will be needed^59^. Alternatively, self-reported scales, such as the Acquired Capability for Suicide Scale^60^ which directly assess suicide intent or possibility, might be able to accurately identify suicide.

This suggests—excluding the limitations of experiments using self-reported scales—that LDAEP might not be a biomarker for suicidal behaviors. Although impulsive-aggressive traits^61,62^ have been associated with MDD patients with suicidal behaviors, often depressed patients had traits of impulsivity and aggression^63^. Despite various previous studies having suggested that LDAEP might have an association with emotional sensitivity, atypical depression symptoms, and impulsivity^22,64,65^; yet, it might not be a possible biomarker for suicide but for characteristics of MDD. In this regard, the role of LDAEP in depression might differ depending on the presence of bipolarity or atypical depressive symptoms^52^, which need to be investigated^34^. Moreover, it is likely that data-driven approaches to psychiatric diagnostics have recently gained more popularity due to the growing realization that the identification of better-specified phenotypes of more homogeneous patient subgroups or subtypes could improve our understanding of patient-specific etiological mechanisms^66,67^. Depending on the depression subtype, there may be changes in EEG according to SA, which could affect our non-significant group difference. Further research should consider the subtypes of depression.

This study had a few limitations. First, since it was cross-sectional in nature, a longitudinal design study would be needed to further analyze the dynamic changes of serotonergic activity in the human brain. Second, various psychological scales were assessed using self-reported measures. Despite the self-reported scales in this study having quite good stability and validity, they were unable to reflect the neural/cognitive basis of clinical characteristics such as impulsivity or emotional regulation. Third, our results might be generalized to patients with MDD. To address these limitations, additional studies are needed in the future.

Despite the foregoing limitations, to the best of our knowledge, this study was the first to compare possible variations in LDAEP among depressed patients with SA and SI, and healthy controls. It also evaluated LDAEP immediately after a SA in a relatively large sample, including unmedicated patients. Its results suggest that LDAEP might not reflect SA in MDD patients, but other characteristics of depression. Future studies should explore which characteristics of depression could be related to LDAEP.

## Methods

### Participants

The participants (N = 130) were enrolled from September 2017 to March 2020. Of these, 49 MDD Patients with SI (23 men and 26 women with a mean age of 32.06 ± 9.34 years) were recruited from the Psychiatry department’s outpatient clinic after a psychiatric interview, while 45 MDD patients with SA (20 men and 25 women with a mean age of 32.07 ± 9.49 years), were referred by the Soonchunhyang Cheonan Hospital’s Medical/Emergency department after fatal SA such as drug intoxication, wrist cutting, hanging or falling down. The patient groups were diagnosed using the Structured Clinical Interview for Diagnostic and Statistical Manual of Mental Disorders, 4th edition for Axis I Psychiatric Disorders^68^, and the Beck’s Suicide Ideation Scale^52^ was used to confirm the suicide intent of depressed patients with SI. Among these 94 depressed patients, 15 had a comorbid anxiety disorder. The 36 healthy controls (19 men and 17 women with a mean age of 31.44 ± 5.20 years) were recruited from the local community through posters. During the initial screening interviews, those with a smoking history of two years were excluded. None of these patients had neurological disorders, severe medical illness, mental retardation, electroconvulsive therapy, alcohol abuse, or head injury. All the depressed patients were drug-naïve and those with SA were evaluated via EEG within 7 days of their SA. All the participants had normal hearing ability confirmed by the 512-Hz tuning fork test^69^ and were right-handed.

### Assessment

Depressive and anxiety symptoms were evaluated using the BDI^70^ and the BAI^71^, respectively. The BDI is a validated scale composed of 21 items for measuring the severity of depressive symptoms^70^. Each BDI question was scored from 0 to 3, with higher scores indicating greater severity of depressive symptoms. The BAI, which consists of 21 items scored on a Likert scale ranging from 0 to 3, and raw scores ranging from 0 to 63^71^ is an anxiety scale that measures the intensity of cognitive, affective, and somatic anxious symptoms experienced during the last 7 days^71^.

To assess impulsivity-related traits, BIS-11 was used^72^. It consists of 11 questionnaires and is designed to assess the personality/behavioral construct of impulsiveness. It also includes three second-order factors (attentional, motor, and non-planning impulsiveness)^72^. DERS is a self-reported scale composed of 36 items for measuring emotional regulation difficulties^73^. Each DERS question was scored from 1 to 5, with higher scores indicating greater difficulties in emotional regulation^73^.

### Data acquisition and analysis

During the EEG task, each participant was tested in a sound-attenuated EEG room. The EEG was acquired using a NeuroScanSynAmps amplifier (Compumedics USA, E1 Paso, TX, USA) with 62 Ag–AgCl electrodes mounted on a Quik Cap using an extended 10–20 placement scheme. The ground electrode was located on the forehead, and the physically linked reference electrode was attached to both mastoids. The vertical electrooculogram (EOG) was positioned above and below the left eye, while the horizontal EOG was placed at the outer canthus of each eye. The impedance was maintained below 5 kΩ. All data were processed with a 0.1–100 Hz band-pass filter and sampled at 1000 Hz. The EEG acquisition procedure was described in our previous study^74,75^.

Recorded EEG data were preprocessed using the CURRY 8 X Data Acquisition package. EEG data was re-referenced to an average reference. Gross artifacts were rejected by a trained person via visual inspection without prior information regarding the origin of the data. Artifacts related to eye movement or eye blinks were eliminated using the mathematical operations in the preprocessing software^52^. Data were filtered using a 0.1–30 Hz band-pass filter and epoched from 100 ms pre-stimulus to 600 ms post-stimulus. These epochs were subtracted from the average value of the pre-stimulus interval for baseline correction. If any remaining epochs contained significant physiological artifacts (amplitude exceeding ± 75 μV) in any of the 62 electrode sites, they were excluded from further analysis. For event-related potential analysis only artifact-free epochs were averaged across trials and participants. The procedure for the preprocessing of EEG was based on our previous study^22^.

### LDAEP

LDAEP was measured as a change in the amplitude of the evoked N1/P2 component in response to different intensities of auditory stimulus^76^. Auditory stimulation contained 500 stimuli with an inter-stimulus interval that was randomized between 500 and 900 ms. Tones of 1000 Hz and 80 ms duration were presented at five intensities (60, 70, 80, 90, and 100 dB SPL) through MDR-D777 headphones (Sony, Tokyo, Japan). These stimuli were generated with E-Prime software (Psychology Software Tools, Pittsburgh, PA, USA). The procedure for the display of the auditory stimulus was based on our previous study^22^.

From the stimulus at the Fz, FCz, and Cz electrodes, the N1 and P2 mean amplitudes between 100–80 ms and 180–300 ms, respectively, were extracted for each subject from the five sound intensities. Thereafter, the mean N1 amplitude was subtracted from the mean P2 amplitude for each of the five stimulus intensities, and the LDAEP was calculated as the slope of the linear regression.

The number of LDAEP epochs used for the analysis did not significantly differ among the MDD patients with SA and SI and the healthy controls (60 dB: 79.17 ± 13.75 vs. 78.97 ± 11.39 vs 77.67 ± 13.53, p = 0.77, 70 dB: 79.67 ± 13.94 vs. 79.05 ± 11.67 vs 78.50 ± 12.97, p = 0.87, 80 dB: 79.53 ± 13.76 vs. 79.53 ± 12.27 vs 77.67 ± 14.05, p = 0.65, 90 dB: 78.87 ± 13.67 vs. 79.73 ± 12.27 vs 77.52 ± 14.17, p = 0.63, 100 dB = 78.63 ± 13.58 vs. 79.88 ± 12.07 vs 77.33 ± 13.04, p = 0.52; SA vs SI vs healthy controls, respectively).

### Statistical analyses

A chi-squared analysis was used for categorical data. Analysis of variance (ANOVA) was used to examine differences in demographic and clinical symptoms among the three groups of subjects. ANOVA with age, gender, and education as covariates was carried out to compare LDAEP slopes at the Fz, FCz, and Cz electrodes among the three groups^16^. Although there was no statistically significant difference in age and gender, both these were controlled as covariates in this study because the previous study had reported that they might affect the amplitude of LDAEP^16^. A least significant difference post-hoc test was used. Relationships between the variables in participants were determined using Pearson’s correlation analysis with a 5,000-bootstrap resampling technique to correct for multiple correlations. Although Bonferroni correction is a strict and needlessly conservative method to avoid errors involving multiple tests, it might result in inappropriately lower *p* values^77^. Bootstrap test for resolving multiple comparison errors is a weaker method than the Bonferroni test, but its robustness and stability have been recognized in various previous studies^78–80^, and it has been widely used in EEG analysis^22,81^. The significance level was set at *p* < 0.05 (two-tailed). All statistical analyses were performed using SPSS 21 (SPSS, Inc., Chicago, IL, USA).

### Ethical approval and informed consent

This study and all experimental protocols were approved by the Soonchunhyang University Cheonan Hospital’s Institutional Review Board and Ethics Committee (approval number: 2017-06-035). The study was also performed in accordance with the approved guidelines, and informed consent was obtained from all the participants.

## Data availability statement

All the authors agreed to make materials, data, and associated protocols promptly available to readers without undue qualifications in material transfer agreements.
